# Profiling non-tuberculous mycobacteria in an Asian setting: characteristics and clinical outcomes of hospitalized patients in Singapore

**DOI:** 10.1186/s12890-018-0637-1

**Published:** 2018-05-22

**Authors:** Albert Y. H. Lim, Sanjay H. Chotirmall, Eric T. K. Fok, Akash Verma, Partha P. De, Soon Keng Goh, Ser Hon Puah, Daryl E. L. Goh, John A. Abisheganaden

**Affiliations:** 1 Department of Respiratory and Critical Care Medicine, Tan Tock Seng Hospital, 11 Jalan Tan Tock Seng, Singapore, 308433 Singapore; 2 Lee Kong Chian School of Medicine, Translational Respiratory Research laboratory, Nanyang Technological University, Clinical Sciences Building, 11 Mandalay Road, Singapore, 308232 Singapore; 3 Yong Loo Lin School of Medicine, National University of Singapore, 12 Science Drive 2, Singapore, 117549 Singapore; 4 Department of Laboratory Medicine, Tan Tock Seng Hospital, 11 Jalan Tan Tock Seng, Singapore, 308433 Singapore; 5 Dalhousie Medical School, Dalhousie University, 1459 Oxford Street, Halifax, NS B3H 4R2 Canada

**Keywords:** Non-tuberculous mycobacteria, Bronchiectasis, Tuberculosis, Epidemiology, *Mycobacterium abscessus*

## Abstract

**Background:**

Non-tuberculous mycobacteria (NTM) infection is an increasing problem worldwide. The epidemiology of NTM in most Asian countries is unknown. This study investigated the epidemiology, and clinical profile of inpatients in whom NTM was isolated from various anatomical sites in a Singaporean population attending a major tertiary referral centre.

**Methods:**

Demographic profile, clinical data, and characteristics of patients hospitalized with NTM isolates at a major tertiary hospital over two-year period were prospectively assessed (2011–2012). Data collected included patient demographics, ethnicity, smoking status, co-morbidities, NTM species, intensive care unit (ICU) treatment, and mortality.

**Results:**

A total of 485 patients (62.1% male) with 560 hospital admissions were analysed. The median patient age was 70 years. Thirteen different NTM species were isolated from this cohort. *Mycobacterium abscessus (M. abscessus)* (38.4%) was most frequently isolated followed by *Mycobacterium fortuitum (M. fortuitum)* (16.6%), *Mycobacterium avium complex* (*MAC)* (16.3%), *Mycobacterium kansasii (M. kansasii)* (15.4%), and *Mycobacterium gordonae (M. gordonae)* (6.8%). Most (91%) NTM was isolated from the respiratory tract. The three most common non-pulmonary sites were; blood (2.7%), skin wounds and abscesses (2.1%), and gastric aspirates (1.1%). A third (34.4%) of the study population had prior pulmonary tuberculosis (PTB). There was a significant association between isolated NTM species, and patient age (*p* = 0.0002). Eleven (2.2%) patients received intensive care unit (ICU) treatment during the study period and all cause mortality within 1 year of the study was 16.9% (*n* = 82). Of these, 72 (87.8%) patients died of pulmonary causes.

**Conclusions:**

The profile of NTM species in Singapore is unique. *M. abscessus* is the commonest NTM isolated, with a higher prevalence in males, and in the elderly. High NTM prevalence is associated with high rates of prior PTB in our cohort.

## Background

Non-tuberculous mycobacteria (NTM) are ubiquitous environmental organisms particularly in water and soil [1]. Their survival in water drainage, hospital water systems, and haemodialysis centres are attributable to its inherent resistance to high temperatures, low pH, and antibiotics [2–4].

NTM Infections are increasing exponentially in their global prevalence, morbidity and mortality [5]. The trend is partially attributed to the availability of improved molecular diagnostic testing [6], improved physicians’ awareness, and a greater number of susceptible hosts. NTM incidence ranges between 7.2 and 13.6 per 100,000 persons [7, 8]. It is however difficult to determine the prevalence and incidence of infection accurately as its isolation microbiologically does not always equate to or even indicate clinical infection.

A wide spectrum of NTM infections is reported including pulmonary, bone, eye, ear, and that affecting the central nervous system. Lymphadenitis, skin abscesses and disseminated infection, the latter in immuno-compromised individuals are also described [9]. The risk factors for NTM infections are varied and include genetic susceptibility, structural lung damage, autoimmune disease, acquired immunodeficiency states including AIDS, malignancy, and solid organ transplants [10]. Use of immunosuppressive drugs such as tumour necrosis factor (TNF) –α blockers also predispose to NTM infection [11, 12].

It is reported that more than 90% of the NTM positive cultures are pulmonary in origin [13]. The reported prevalence of NTM pulmonary infections in the USA varies between 4.1 and 14.1 per 100,000 [14]. NTM pulmonary infections are most common in females and those older than 65. Geographic and ethnic variations are also observed with NTM pulmonary infection [15]. For instance, in a study of NTM species from respiratory specimens of 20,182 patients in 30 countries across six continents, the most common NTM species identified was *Mycobacterium avium complex* (*MAC*), followed by *Mycobacterium gordonae (M. gordonae*), *Mycobacterium xenopi* (*M. xenopi)* and *Mycobacterium kansasii* (*M. kansasii*) [16]. In the same study, it was noted that although *MAC* is the commonest overall, its prevalence is higher in Asian countries [16]. In Singapore, a key South-East Asian island state, the distribution of NTM species is largely unknown and hence we studied the NTM profiles, clinical characteristics and outcomes in a large inpatient Asian cohort attending a major tertiary referral centre.

## Methods

This prospective observational study included all adult patients where NTM was isolated on at least one specimen during a hospital admission at Tan Tock Seng Hospital, Singapore between January 2011 and December 2012 (2-year period). Patient demographics, ethnicity, smoking status, co-morbidities, and NTM species isolated were obtained from the computerized patient support system (CPSS) and collated for analysis. All data complied with the Singapore Personal Data Protection Act (PDPA) 2012 and the Institutional Review Board (IRB) of the National Healthcare Group approved the study protocol.

NTM specimens from pulmonary and non-pulmonary sites were analysed. The specimens obtained from ‘pulmonary sites’ included sputum, bronchoalveolar lavage (BAL), pleural biopsies and fluid. The ‘non-pulmonary’ site specimens included skin abscess fluid, skin wound swabs, blood, urine, and bone biopsy specimens. All specimens were stained by the Ziehl-Neelsen method according to the American Thoracic Society guidelines [17]. *Mycobacterium tuberculosis* (MTB) and NTM isolates were distinguished by their growth rate, colonial morphology, pigmentation, and by negative DNA probe (AccuProbe; Gen-Probe Inc., San Diego, CA) and NAP (ρ-nitro-α-acetylamino-β-hydroxy-propiophenone) tests for MTB. NTM species were identified by DNA reverse hybridization (INNO-LiPA MYCOBACTERIA v2, Innogenetics NV, Ghent, Belgium) and high-performance liquid chromatography. Further identifications were performed by 16S ribosomal RNA sequencing using primers 16S-27F (5′-AGA GTT TGA TCM TGG CTC AG-3′) and 16S-907R (5’-CCG TCA ATT CMT TTR AGT TT-3′).

Patient demographics are presented as summary statistics. Categorical variables were compared using Chi-squared analysis or Fisher’s exact test. If continuous data were normally distributed, unpaired t-tests were used, and if non-normal, the Mann-Whitey rank sum test employed. For all statistical analysis, *p* < 0.05 was considered statistically significant.

## Results

### Characteristics of the study population

A total of 485 adult patients (62% male) with 560 NTM isolates were studied. The median (IQR) age and body mass index (BMI) of the study population were 70 (58–82) years and 19 (16–23) respectively. The majority were of Chinese descent (82%), followed by Malay (8%), Indian (4%) and other ethnicities including Eurasians (6%). Ninety five (19.5%) were current smokers. Bronchiectasis (28.7%) was the commonest underlying pulmonary disease, followed by chronic obstructive pulmonary disease (COPD) (14.2%). The three commonest non-pulmonary co-morbidities were hypertension (32.2%), hyperlipidaemia (25.8%), and diabetes mellitus (17.9%). Fifty two (10.7%) had human immunodeficiency virus (HIV) infection (Table 1).

**Table 1:** Demographics and clinical characteristics of the study population (*n* = 485)

Age (years): median (IQR)	70 (58–82)
Gender (male): n (%)	301 (62.1%)
BMI (kg/m^2^): median (IQR)	19.4(16.4–23)
Smoking status: n (%)	
Current smoker	95 (19.6%)
Ex- smoker	94 (19.4%)
Never	296 (61%)
Previous history of PTB: n (%)	167 (34.4%)
Active PTB: n (%)	3 (0.6%)
Co-morbidities (pulmonary): n (%)	
Bronchiectasis	139 (28.7%)
COPD	69 (14.2%)
Asthma	32 (6.6%)
Pulmonary fibrosis	18 (3.7%)
Co-morbidities (non- pulmonary): n (%)	
Hypertension	156 (32.2%)
Hyperlipidaemia	125 (25.8%)
Diabetes mellitus	87 (17.9%)
Coronary artery disease	63 (12.9%)
HIV infection	52 (10.7%)
Psychiatry disorder	38 (7.8%)
Malignancy	29 (6%)
Cerebrovascular disease	23 (4.7%)
Chronic renal impairment	16 (3.3%)
Rheumatoid arthritis	11 (2.2%)
Prevalence of isolates per 100,000 hospital admissions	511/100,000

### NTM species and isolation sites

A total of 13 species of NTM were identified in this study. The five most frequently isolated NTM were *M. abscessus* (215 isolates; 38.4%), *M. fortuitum* (93 isolates; 16.6%), *MAC* (91 isolates; 16.3%), *M. kansasii* (86 isolates; 15.4%), and *M. gordonae* (38 isolates; 6.8%). These five species accounted for 93.5% of all NTM species isolated (Table 2). Five hundred and eleven (91%) of all the NTM isolates were from pulmonary sites. The three most common non-pulmonary sites were; blood specimens (15 isolates; 2.7%), skin wounds and abscesses (12 isolates; 2.1%), and gastric aspirates (6 isolates; 1.1%) (Table 3). *M. abscessus* was the most frequently isolated NTM species from pulmonary specimens (202 isolates; 39.5%), whilst *MAC* (14 isolates; 28.6%) was the most frequently NTM species isolated from non-pulmonary specimens, closely followed by *M. abscessus* (13 isolates; 26.5%) (Table 3).

**Table 2:** Frequency of NTM species isolated during the study period (*n* = 650)

NTM species	N (%)
*Mycobacterium abscessus*	215 (38.4%)
*Mycobacterium fortuitum complex*	93 (16.6%)
*Mycobacterium avium complex*	91 (16.3%)
*Mycobacterium kansasii*	86 (15.4%)
*Mycobacterium gordonae*	38 (6.8%)
*Mycobacterium chelonae*	9 (1.6%)
*Mycobacterium lentiflavum*	8 (1.4%)
*Mycobacterium scrofulaceum*	6 (1.0%)
*Mycobacterium haemophilum*	4 (0.7%)
*Mycobacterium simiae*	3 (0.5%)
*Mycobacterium szulgai*	3 (0.5%)
*Mycobacterium terrae complex*	2 (0.4%)
*Mycobacterium mucogenicum*	2 (0.4%)

**Table 3:** Distribution of NTM species by isolation sites

	Pulmonary sites (*n* = 511)	Skin and soft tissues (*n* = 12)	Blood (*n* = 15)	Gastric aspirate (*n* = 6)	Urine (*n* = 4)	Liver (*n* = 4)	Other (*n* = 8)
*M. abscessus* – n (%)	202(39.5%)	7 (58%)	2 (13.3%)	3 (50%)	1 (25%)	0 (0)	0 (0)
*M. fortuitum* – n (%)	85 (16.6%)	1 (8.3%)	0 (0)	0 (0)	2 (50%)	2 (50%)	3 (37.5%)
*M. avium* – n (%)	77 (15.1%)	0 (0)	10 (66.7%)	0 (0)	1 (25%)	0 (0)	3 (37.5%)
*M. kansasii* – n (%)	79 (15.5%)	2 (16.7%)	1 (6.7%)	1 (16.7%)	0 (0)	2 (50%)	1 (12.5%)
*M. gordonae* – n (%)	36 (7%)	0 (0)	0 (0)	2 (33.3%)	0 (0)	0 (0)	0 (0)
*M. chelone* – n (%)	9 (1.8%)	0 (0)	0 (0)	0 (0)	0 (0)	0 (0)	0 (0)
*M. lentifalvum* – n (%)	8 (1.6%)	0 (0)	0 (0)	0 (0)	0 (0)	0 (0)	0 (0)
*M. scrolaceum* – n (%)	6 (1.2%)	0 (0)	0 (0)	0 (0)	0 (0)	0 (0)	0 (0)
*M. haemophilum* – n (%)	0 (0)	2 (16.6%)	1 (6.7%)	0 (0)	0 (0)	0 (0)	1 (0)
*M. szulgai* – n (%)	3 (0.6%)	0 (0)	0 (0)	0 (0)	0 (0)	0 (0)	0 (0)
*M. simiae* – n (%)	3 (0.6%)	0 (0)	0 (0)	0 (0)	0 (0)	0 (0)	0 (0)
*M. terrae* – n (%)	2 (0.4%)	0 (0)	0 (0)	0 (0)	0 (0)	0 (0)	0 (0)
*M.mucogenicum- n (%)*	1 (0.2%)	0 (0)	1 (6.7%)	0 (0)	0 (0)	0 (0)	0 (0)

### Association of gender, age, and NTM isolates

Most NTM isolates were found in male patients and those aged above 50 years (Table 4). There was a significant association between NTM isolation and patients’ age (*p* = 0.0002). On subgroup analysis, *M. abscessus* (81.8%), *M. fortuitum* (96.3%), and *MAC* (84.5%) were commonly isolated from patients aged above 50. Contrary to the other species, *M. abscessus* was the only NTM species to be identified in younger patients (below age 30) (13 patients; 6%) (Table 4). There was no statistically significant difference noted among the NTM species isolated across the other age groups (Table 4).

**Table 4:** Prevalence of NTM isolates by gender and age

	*M. abscessus* (*n* = 195)	*M. fortuitum* (*n* = 83)	*M.* *avium* (*n* = 78)	Other NTM (*n* = 129)	*M.* *fortuitum*	*M.* *avium*	Other NTM
	N (%)				OR (95%CI)		
Male	120 (62)	45(54)	54 (69)	82 (64)	0.74 (0.44–1.24)	1.40 (0.80–2.46)	1.09 (0.6–1.73)
Age (years)							
< 30	10 (5)	0 (0)	0 (0)	0 (0)	0.11 (0.01–1.83)^a^	0.11 (0.01–1.95)	0.07 (0.01–1.18)^b^
31–50	25 (13)	5 (6)	9 (12)	17 (13)	0.44 (0.16–1.18)	0.89 (0.39–1.99)	1.03 (0.53–1.99)
51–70	73 (37)	34 (41)	29 (37)	43 (33)	1.16 (0.69–1.96)	0.99 (0.58–1.70)	0.83 (0.52–1.33)
> 70	87 (45)	44 (56)	40 (51)	69 (54)	1.12 (0.68–1.83)	1.31 (0.77–2.21)	1.43 (0.91–2.23)

^a^*M. abscessus* vs. *M. fortuitum*, *p* = 0.04

^b^*M. abscessus* vs. *Other NTM, p* = 0.007

### Pulmonary tuberculosis, bronchiectasis and NTM isolates

Three patients were found to have co-infection with pulmonary tuberculosis (PTB) and *M. abscessus* during the study period. A third (34.4%) of the study population had prior PTB. Previous PTB accounted for the underlying aetiology in 88 (18.1%) of those with bronchiectasis described in this cohort. Among patients with bronchiectasis (*n* = 139), the three commonest NTM isolates were *M. abscessus* (41.7%; 58/139), *MAC* (15.8%, 22/139), and *M. fortuitum* (12.9%, 18/139). There was a preponderance for NTM in females in patients with bronchiectasis (52% vs. 32.4% respectively, *p* < 0.0001) in contrast to the male preference overall as reported above.

### Intensive care treatment, mortality and NTM isolates

Eleven (2.2%) patients received intensive care unit (ICU) treatment during the study period and 82 (16.9%) patients died during the course of the study. Of these deaths, 72 (87.8%) were due to pulmonary causes. *M. abscessus* was the most frequently isolated NTM species in those who required ICU treatment or died (Table 5).

**Table 5:** Intensive care unit treatment, mortality and NTM isolates

	Overall event rate	Species				
		*M. abscessus*	*M. kansasii*	*MAC*	*M. fortuitum*	*M. gordonae*
ICU admissions	11 (2.2%)	3 (27.3%)	3 (27.3%)	2 (18.2%)	2 (18.2%)	1 (9%)
Mortality						
- All cause	82 (16.9%)	38 (46.3%)	13 (15.9%)	17 (20.7%)	6 (7.3%)	3 (3.7%)
- Pulmonary cause	72 (14.8%)	35 (48.6%)	13 (18.1%)	14 (19.4%)	5 (6.9%)	2 (2.8%)

## Discussion

We demonstrate a unique profile of NTM species in Singapore, a Southeast Asian city state. *M. abscessus* was the commonest NTM isolated, with a higher prevalence in males and the elderly. We further illustrated a high prevalence of NTM in hospitalised patients. Most significantly however, *M. abscessus* was the commonest of the 13 NTM species isolated and accounted for approximately a third of all the isolates followed by *M. fortuitum* (16.6%) and *MAC* (16.3%). This suggests a unique Asian profile in the spectrum of isolated NTM from hospitalised inpatients. Furthermore, half (53%) of our study population had an underlying pulmonary disorder with bronchiectasis being most common (28.7%). Interestingly, over one third of the cohort had prior PTB which in a large proportion accounted for the aetiology of their detected bronchiectasis.

In a small Singapore study published more than two decades ago, Teo and Lo [18] found that MAC was the most common NTM isolated. The difference in their NTM profile from ours may be due to their small sample size of the older study. Other factors may be the improvement in laboratory techniques in diagnosing rapidly growing mycobacteria (RGM) over the intervening period as well as the emergence of RGM, especially *M. abscessus* as a human pathogen in the region.

NTM related disease has gained much attention due to its increasing prevalence globally and the improved ability for its isolation [15, 19–21]. There is geographic variation in the NTM species isolated with similar differences observed in disease manifestations [15, 16, 22]. *MAC* has been reported as the commonest NTM species isolated worldwide, followed by *M. gordonae*, and *M. xenopi* [16]. In the case of pulmonary NTM disease while *MAC* is generally the universal preponderant species, there is much regional variation with regard to other commonly encountered NTM species; in the USA and Japan *M. kansasii* is the next most common species, in South Korea it is *M. abscessus*, and in France *M. Xenopi* [17, 23–26].

Our finding of predominant *M. abscessus* is novel and likely unique in an East Asian context. The existing literature includes a recent study on 20,182 patients by the NTM-Network European Trials Group (NET) who reported that *MAC* species were the commonest (47%) NTM species isolated worldwide [16]. In the same study, RGM such as *M. abscessus* and *M. fortuitum* made up only 10–20% of all the NTM isolates described, in contrast to our dataset. Interestingly however, these isolates originated predominantly from Asian countries including Taiwan and South Korea. We believe our NTM profile is unique, dominated with *M. abscessus* followed by *M. fortuitum* and *MAC*. Likely reasons for the high prevalence of *M. abscessus* in our study population remains unclear, however geographical, climatic, host and genetic factors have all been previously proposed [27].

There are a number of potential explanations for our findings in relation to past PTB in one third of those where NTM was isolated. Firstly, the incidence of TB is modest between 35 to 45 cases per 100,000 in Singapore. PTB also causes structural and functional lung damage including bronchiectasis, airway stenosis, bronchovascular distortion, and fibrosis that facilitate NTM growth [28]. Secondly, on Ingenuity Pathway Analysis (IPA) of gene to gene relationships, it is described that focus genes such as the Toll Like Receptor-2 (*TLR2*), Interleukin12 (*IL12*), and Interferon-gamma (*IFNG*) all play important roles in the innate and adaptive immune response and where genetic polymorphisms are present leads to an increased risk of both TB and NTM infection [29]. Thirdly, from network genes analysis, nuclear factor-κB (*NFκB)* complex, ERK1/2, and p38MAPK (mitogen-activated protein kinase) pathways all affect disease survival where both TB and NTM are implicated. Defects in these pathways increase the risk of both TB and NTM infections [30–32]. Prior studies also demonstrate that vitamin deficiencies (e.g. vitamins A, B, C, D, E, K, and lycopene) alter immune regulation and increase the risk of mycobacterial infection. Vitamin deficiencies are common in the Asian population and could explain our findings of high PTB and NTM prevalence [29].

In a study of 2548 patents with pulmonary NTM in the USA, Adjemain et al. found a higher prevalence of pulmonary NTM in the Asian and Pacific Islander population with a male predominance, in contrast with the white population where the prevalence is lower and female patients form the predominant group [15]. This preponderance of male patients infected with NTM disease is similarly seen in our study population which comprises various Asian ethnicities. In their work, the interactions of genetic, behavioural and environmental factors were cited as possible causes for this difference in the prevalence between ethnic groups.

In our study we found a high prevalence of NTM isolates among the elderly (median age 70 years); this is consistent with recent reports from USA and South Korea [19, 25]. Ageing and the increased incidence of co-morbidities in the elderly may play an important role in the acquisition and subsequent persistence of NTM. NTM isolation increases with age and most of these developed pulmonary NTM [25, 27]. As the population of Singapore aged 65 and above is expected to double by 2030, it is important to recognise NTM infection and particularly its association with age as an important public health issue with potential significant consequences for affected patients and use of healthcare resources. An important shortcoming of this study is the study of the profile of NTM isolates over a 2 year period which may not be long enough to determine associated trends particularly the development of active NTM pulmonary infection. Follow-up studies over longer periods may overcome this limitation, and likely provide additional and important information on trend and factors that may predict onset of active NTM pulmonary infection. Additionally, we only curated a number of clinical variables in this Asian-based assessment of NTM and lacked a comparator non-Asian group. Addressing these in future studies will likely provide a more comprehensive dataset permitting a greater degree of clinical relevance in the Asian context to be established.

The clinical outcomes of our study population were poor, further highlighting the importance of recognising NTM infection. Likely explanations for the poor prognosis include older patient age and associated co-morbidities and the predominance of *M. abscessus,* which in itself is associated with multi-drug resistance and accelerated declines in lung function [33].

## Conclusion

In summary, our study demonstrates a unique profile of NTM species in Singapore. *M. abscessus* was the commonest NTM isolated, with higher prevalence in males and the elderly. A high prevalence of NTM is associated with a high burden of past PTB likely explained by PTB related pulmonary damage thus increasing the risk of acquiring NTM, or alternatively a strong genetic susceptibility to both MTB and NTM infection amongst the Asian population. In addition, the higher prevalence of NTM infection among our older population is significant particularly in the context of the anticipated rise in global ageing which necessitates the need to better understand epidemiological trends, clinical consequences, and economical burden of NTM infection across both Asia and internationally.

## Abbreviations

AIDS: Acquired immunodeficiency syndrome
CPSS: Computerized patient support system
HIV: Human immunodeficiency virus
ICU: Intensive care unit
*IFNG*: Interferon-gamma
*IL12*: Interleukin 12
IPA: Ingenuity pathway analysis
IRB: Institutional Review Board
*MAC*: *M. avium complex*
NTM: Nontuberculous mycobacteria
PDPA: Personal Data Protection Act
PTB: Pulmonary tuberculosis
RGM: Rapidly growing mycobacteria
*TLR2*: Toll like receptor gene
TNF: Tumour necrosis factor
